# Crystal Structure of *Mycobacterium tuberculosis* Elongation Factor G1

**DOI:** 10.3389/fmolb.2021.667638

**Published:** 2021-09-03

**Authors:** Xiaopan Gao, Xia Yu, Kaixiang Zhu, Bo Qin, Wei Wang, Pu Han, Justyna Aleksandra Wojdyla, Meitian Wang, Sheng Cui

**Affiliations:** ^1^ NHC Key Laboratory of Systems Biology of Pathogens, Institute of Pathogen Biology, And Center for Tuberculosis Research, Chinese Academy of Medical Sciences and Peking Union Medical College, Beijing, China; ^2^ National Clinical Laboratory on Tuberculosis, Beijing Key Laboratory for Drug-resistant Tuberculosis Research Beijing Chest Hospital, Beijing Tuberculosis and Thoracic Tumor Institute, Capital Medical University, Beijing, China; ^3^ CAS Key Laboratory of Pathogenic Microbiology and Immunology, Institute of Microbiology, Chinese Academy of Sciences, Beijing, China; ^4^ Swiss Light Source at the Paul Scherrer Institut, Villigen, Switzerland; ^5^ Sanming Project of Medicine in Shenzhen on Construction of Novel Systematic Network Against Tuberculosis, National Clinical Research Center for Infectious Diseases, Shenzhen Third People’s Hospital, Southern University of Science and Technology, Shenzhen, China

**Keywords:** *Mycobacteria tuberculosis*, Elongatin factor G (EF-G), crystal structure, antituberculosis drug, ribosome-bound EF-G

## Abstract

*Mycobacterium tuberculosis* (*Mtb*) caused an estimated 10 million cases of tuberculosis and 1.2 million deaths in 2019 globally. The increasing emergence of multidrug-resistant and extensively drug-resistant *Mtb* is becoming a public health threat worldwide and makes the identification of anti-*Mtb* drug targets urgent. Elongation factor G (EF-G) is involved in tRNA translocation on ribosomes during protein translation. Therefore, EF-G is a major focus of structural analysis and a valuable drug target of antibiotics. However, the crystal structure of *Mtb* EF-G1 is not yet available, and this has limited the design of inhibitors. Here, we report the crystal structure of Mtb EF-G1 in complex with GDP. The unique crystal form of the Mtb EF-G1-GDP complex provides an excellent platform for fragment-based screening using a crystallographic approach. Our findings provide a structure-based explanation for GDP recognition, and facilitate the identification of EF-G1 inhibitors with potential interest in the context of drug discovery.

## Introduction

*Mycobacterium tuberculosis* (*Mtb)*, one of the deadliest bacterial pathogens causing tuberculosis, is still threatening humanity in the 21^st^ century. According to the World Health Organization (WHO) global tuberculosis report, this single pathogen claimed nearly 1.2 million lives worldwide in 2019 (https://www.who.int/tb/publications/global_report/en/). The emergence of multidrug-resistant tuberculosis (MDR-TB) and extensively drug-resistant tuberculosis (XDR-TB) undermine conventional treatments. For this reason, it is urgent to identify novel inhibitor targets and develop new and effective Mtb drugs.

Elongation factor G (EF-G) is a highly conserved and essential GTPase protein that catalyzes tRNA translocation on ribosomes at several stages of protein synthesis. In addition, EF-G is involved in ribosome recycling and suppression of ribosomal frameshifting (Rodnina et al., 2019). Therefore, EF-G represents a promising target for drug design. Fusidic acid (FA), a well-known drug, is widely used in the clinic against EF-G in Gram-positive bacteria (Fernandes, 2016). FA also exhibits antibacterial activity against Mtb *in vitro* (Cicek-Saydam et al., 2001). FA inhibits protein synthesis by binding to EF-G and preventing its release after GTP hydrolysis and translocation (Akinpelu et al., 2020). In addition, argyins exhibiting antibacterial activity against Gram-negative pathogens also target EF-G via a novel mechanism (Nyfeler et al., 2012; Jones et al., 2017).

Structural characterization of Mtb EF-G1 has been limited by the lack of a Mtb EF-G1 crystal, and this has hampered structure-based inhibitor design. Although FA is ineffective against Mtb *in vivo* owning to pharmacokinetic limitations, as demonstrated using a rodent model of Mtb characterized by rapid clearance and poor exposure (Kigondu et al., 2014; Njoroge et al., 2019), hence repurposing the idea that FA and its derivatives could represent a potential novel strategy for TB drug discovery (Njoroge et al., 2019). Indeed, various FA derivatives have been proposed as anti-TB agents (Kigondu et al., 2014; Dziwornu et al., 2019; Njoroge et al., 2019; Strydom et al., 2020). The structure determination of Mtb EF-G1 represents the key step for the structure-based design of inhibitors, in future activities, which will be investigated to assess their potential in the context of anti-TB drug development.

In this study, we purified recombinantly expressed Mtb EF-G1 and characterized its GDP binding activity. We further present the first crystal structure of Mtb EF-G1 in complex with GDP. The Mtb EF-G1●GDP crystal structure presents a complete description of GDP recognition and provides solid structural insight into the design and development inhibitors targeting Mtb EF-G1.

## Materials and Methods

### Plasmid Construction, Protein Expression, and Purification

The gene encoding Mtb EF-G1 was amplified from the genomic DNA of the Mtb H37RV strain by PCR. The PCR product was then cloned into the expression vector, pCOATexp, derived from pTYB1 vector (New England Biolabs) as previously described (Srivastava et al., 2016), expressing Mtb EF-G1 with a C-terminal His tag. The sequence of the constructed plasmid was verified by DNA sequencing. For protein expression and production, the plasmids encoding Mtb EF-G1 with a C-terminal His tag was transformed in to *E. coli* BL21 (DE3) competent cells. A single clone was randomly picked from a LB plate and inoculated in LB medium at 37°C and protein expression was induced by the addition of 0.5 mM isopropyl β-D-1-thiogalactopyranoside (IPTG) when the culture OD_600_ reached approximately 1.0. the bacterial culture was cooled to 22°C in incubator and continued with shaking speed of 180rpm overnight after induction. Cells were harvested and washed three times with lysis buffer (20 mM Tris-HCl, pH 8.0,150 mM NaCl, 10 mM imidazole, 10 mM β-mercaptoethanol, 1 mM PMSF) by centrifugation and lysed sonication in lysis buffer. Cells debris was removed after centrifugation at 16000g for 1 h at 4°C and the supernatant was filtered through a 0.45 μm syringe filter, and applied to Ni-NTA resin (Qiagen) pre-equilibrated with lysis buffer. The resin was washed with 10 column volumes of washing buffer (20 mM Tris-HCl, pH 8.0,150 mM NaCl, 20 mM imidazole, 10 mM β-mercaptoethanol, 1 mM PMSF) to remove unbound and nonspecifically bound proteins. The targeted protein was eluted with elution buffer containing 300 mM imidazole. The his-tagged Mtb EF-G1 was loaded into a 5 ml anion exchange column Hitrap Q HP column (GE Healthcare) and eluted with a linear gradient of 75–1,000 mM NaCl. The eluted protein was collected and concentrated and was further purified using a Superdex 200 column pre-equilibrated with a buffer (20 mM Tris-HCl, pH 8.0,150 mM NaCl). The purified protein was collected, concentrated using AmiconUltra-15 with 10 kDa cut-off (Millipore) to 10 mg/ml before crystallization trials.

When preparing the selenomethionine-substituted Mtb EF-G1 for crystallization, the encoding gene was expressed in the *E.coli* methionine auxotrophic strain B834 (DE3) and cultured in media composed of SelenoMet Medium Base Mix (Molecular Dimensions) supplemented with L-selenomethionine (Sigma). The purification procedure for the SeMet derivative was the same as described above for the native protein, except that the buffers were supplemented with 2 mM DTT.

### Crystallization

Crystallization trials of Mtb EF-G1 in complex with GDP were conducted in a hanging-drop vapor-diffusion system at 18◦C. Briefly, the Mtb EF-G1 (10 mg/ ml) was mixed with 5 mM GDP on ice for 30 min before crystallization. The Mtb EF-G1●GDP was crystallized by mixing 1 μL sample and reservoir buffer containing 20 mM magnesium chloride, 50 mM MOPS pH 7.0, 55%TacsimateTM pH 7.0 and 5 mM Hexammine cobalt (III) chloride. Crystals were cryoprotected by soaking crystals in reservoir buffer supplemented with 20% ethylene glycol and flash cooled in liquid nitrogen.

### Data Collection, Phasing, and Structure Determination

Complete datasets were collected at beamline BL17U at the Shanghai Synchrotron Radiation Facility (SSRF), Shanghai, China, and at SLS beamline X06DA at the Swiss Light Source, Paul Scherrer Institut, Villigen, Switzerland. Highly redundant X-ray diffraction data for Mtb EF-G1●GDP crystal containing selenomethionine were collected at a wavelength of 0.97931 Å. The reflections were integrated and processed using XDS (Kabsch, 2010). The structure was determined by the single-wavelength anomalous diffraction (SAD) method. The heavy atom substructure with 18 Se atoms were firstly located by dual-space direct-methods program SHELXC/D from the difference of Friedel pairs in the Se-SAD data (Sheldrick, 2010). The initial phases had a figure of merit (FOM) of 0.444 in the resolution range 47.95–3.30 Å. The intrinsic phase ambiguity was broken by iterative direct-methods SAD phasing rooted in the pipeline Iterative Protein Crystal structure Automatic Solution (IPCAS) (Ding et al., 2020). The iteration control was set as OASIS-DM-AutoBuild/Buccaneer with 15 cycles. For each cycle of this dual-space iterative framework, phases were refined and improved in reciprocal space using OASIS and the real space constrains was applied to electron density map using DM (Cowtan and Main, 1996; Hao et al., 2000). The model was built alternatively between AutoBuild and Buccaneer within the iterations to avoid the premature convergence (Cowtan, 2006; Terwilliger et al., 2008). To further improve the quality of the model and phases, the iterative direct-methods model completion was also conducted through IPCAS under the iteration control as OASIS-DM-AutoBuild/Buccaneer with 10 cycles (He et al., 2007). A partial structure was automatically built with 94.6% of the total residues docked to the sequence after two direct-methods iterations.

Coot and Phenix.refine were subsequently used for final model building and refinement, respectively (Emsley et al., 2010; Echols et al., 2012). The final model was validated by MolProbity Chen V. B. et al. (2010) and showed excellent refinement statistics and stereochemical quality. The detailed statistics of data collection, reduction, and structure refinement are presented in Supplementary Table S1. All structural figures were generated by PyMOL.

### Structure Alignment and Modeling

Structural alignment was performed with PyMOL (http://www.pymol.org) using the corresponding coordinates deposited in the PDB. PyMOL performs a sequence alignment followed by a structural superposition, and then carries out zero or more cycles of refinement in order to minimize the RMSD (Root Mean Square Deviation) between the aligned residues. All structure superposition was achieved by aligning domains I and II of the EF-G structures. The homology model for Mtb EF-G1-FA was carried out by superimposing Mtb EF-G1 onto the structure of EF-G bound to the *T. thermophilus* 70S ribosome with GDP and FA (PDB id: 4V5F) based on domains I and II in PyMOL.

### Isothermal Titration calorimetry

All titration were performed using a MicroCalTM iTC200 calorimeter (MicroCal, United States) at 25◦C as previously described (Gao et al., 2018). Both the protein and the GDP were dissolved in the same buffer (20 mM Tris-HCl, pH = 8.0, 100 mM NaCl) to ensure a reasonable baseline. The concentrations of Mtb EF-G1 was 0.02 mM and GDP was 1 mM. For each titration, 18 injections of 2 μL of titrant were made at a 120-s interval using a stir rate of 600 rpm. Data were subtracted with dilution heat of the ligand from control assay, where GDP was titrated into the buffer. Raw data were integrated and analyzed by nonlinear curve fitting using Origin isothermal titration calorimetry analysis program equipped by Microcal.

### Surface Plasmon Resonance Analysis

The interaction of FA with Mtb EF-G1was assessed by surface plasmon resonance (SPR) using a BiaCore T200 equipped with CM5 sensor chips (GE Healthcare) at 25°C. The surfaces of the sample and reference flow cells were activated as previously described (Chi et al., 2020). The Mtb EF-G1 was diluted in 10 mM sodium acetate buffer, pH 4.5, and immobilized on the chip at 19,000 resonance units (RU). For analysis of FA binding, a series of FA concentrations were passed sequentially over chips immobilized with Mtb EF-G1. The data were fitted with a 1:1 steady-state affinity model using BIA evaluation 1.0 software.

### Size Exclusion Chromatography

Gel-filtration chromatography was conducted using a Superdex 200 Column (GE healthcare) .The column was pre-equilibrated with buffer containing 20 Mm Tris HCl (pH 8.0), 150 mM NaCl and calibrated using molecular weight standards. Purified Mtb EF-G1were fractionated at a flow rate of 0.15 ml/ min.

## Results and Discussion

### Biochemical Characterization of Mtb EF-G1

To provide solid structural insight into the Mtb EF-G1, we first expressed soluble Mtb EF-G1 by performing systematic optimization of the overexpression condition. This included varying the induction temperature (37°C, 22°C, 18°C), the concentration of the IPTG inducing reagent (0.1 mM, 0.5 mM), and the *Escherichia coli* host bacterial strain (BL21 [DE3], Rosetta [DE3]). Finally, we obtained stably expressed Mtb EF-G1 at high yield in *E. coli* BL21 [DE3] with an induction temperature of 22°C and 0.5 mM IPTG (Supplementary Figures S1A,B). To investigate the GDP binding ability of Mtb EF-G1, we first carried out isothermal titration calorimetry (ITC) experiments to verify the GDP affinity of Mtb EF-G1.The results showed that Mtb EF-G1 binds GDP with an apparent dissociation constant (*K*_*d*_) of 11.5 ± 0.3 μM (Supplementary Figure S1C). This *K*_*d*_ value is comparable to the dissociation constant reported for other species EF-G (Martemyanov and Gudkov, 2000). These results indicated that Mtb EF-G1 was correctly folded and is suitable for subsequent crystallography.

### Structure Determination of Mtb EF-G1

The crystals of Mtb EF-G1 diffracted the X-ray to 3 Å. The three-dimensional structure of Mtb EF-G1 was solved by the SAD method. The data collection, structure-refinement, and validation statistics are summarized in Supplementary Table S1. Comparing to several EF-G structures complexed with GDP that crystallized in the P212121 space group (PDB id: 1DAR, 4MYT, and 2BM0) in other species, the current crystal form of Mtb EF-G1 has unique space group C222_1_, larger cell dimensions and unique crystal packing (Supplementary Table S1).

Interestingly, Matthews coefficient calculations indicated the presence of either a dimer (VM = 3.58 Å3/Da, a solvent content of approximately 65.6%) or a trimer (VM = 2.38 Å3/Da,a solvent content of 48.39%) in the asymmetric unit (ASU). The highest probability (0.77) was for three molecules in the ASU based on the resolution of the data (Matthews, 1968; Kantardjieff and Rupp, 2003). However, further crystallographic analysis confirmed that the ASU contained two enzyme molecules with a correct electron density map and reasonable R_work_ (20.6%)/R_free_ (24.1%) values, instead of a crystallographic monomer in the ASU as in other EF-G structures (Supplementary Figures S2A,B).

The unique crystal packing, high solvent space (65.6%), and reasonable resolution (3 Å) of the Mtb EF-G1 crystal form provides several advantages for high-throughput fragment-based screening using a crystallographic approach targeting Mtb EF-G1: (1)Although the active site is occupied by GDP, which likely prevents the screening of inhibitors/fragments, it is still useful for identifying allosteric sites. For example, *Pseudomonas aeruginosa* EF-G1 in complex with argyrin B revealed a novel binding site at the interface of domains III and V that is away from the GDP binding site (PDB id: 4FN5), which supports our assertion; (2) The novel crystal form of Mtb EF-G1 can be produced easily with high repeatability, and has a higher solvent content and looser packing than previously reported crystal forms, which may facilitate rapid diffusion of compounds into crystals during soaking experiments.

### Overall Structure of Mtb EF-G1

**FIGURE 1:**
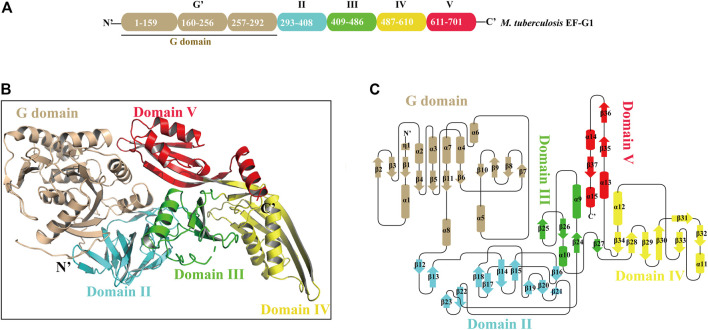
The overall structure of Mtb EF-G1. **(A)** Domain organization of Mtb EF-G1. **(B)** Ribbon model of the Mtb EF-G1 crystal structure. The individual domains are differently colored, with G domain in wheat, domain II in cyan, domain III in green, domain IV in yellow and domain V in red. **(C)** Topology diagram of Mtb EF-G1.The domains are colored the same as in panel **(B)**.

Mtb EF-G1 has five structural domains that fold into an elongated shape with dimensions of 118 Å × 60 Å × 50 Å (Figures 1A,B). The N-terminal G domains are composed of a classic GTPase domain (G domain) and an additional G′ subdomain. The other domains are numbered consecutively based on sequence and structural similarity (II-V; Figure 1A; Supplementary Figure S3). Alignment of the primary sequences of EF-G from different species indicated that Mtb EF-G1 shares higher identity with *Mycobacterium smegmatis* EF-G (85%) and *Arthrobacter globiformis* EF-G (72%) than with *Staphylococcus aureus*, *P. aeruginosa*, *E. coli*, and *Thermococcus thermophilus* EF-G (55–58%; Supplementary Figure S3). Comparison between EF-G1 and another EF-G-like protein in Mtb (FusA2 or EF-G2) revealed a low degree of homology (30%; Supplementary Figure S4). However, Domains I−V are conserved amongst the EF-G1 and EF-G2 sequences. The G domain (residues 2-292) shows great structural similarity with other GTPase family except with an insert of ∼90 residues (residues 160-256). The core of the G domain exhibits an α/ fold with six β-sheets (β1-β5, β11) surrounded by five α helices (α1-α4, α8). The G′ subdomain is comprised of a five-stranded mixed β-sheet (β6-β10) followed by three α-helices (α5-α7), and exhibits βββββααα topology (Figure 1C; Supplemenatry Figure S5A, colored wheat). The “effector loop” or “switch I” region (residues45-65) is largely disordered and is not visible in any of the reported EF-G structures. The G domain, including G1, G2, G3, G4, and G5 motifs, is highly conserved in various organisms (Supplementary Figure S3), and the GTP binding motifs (G1, G3, G4) are also conserved in Mtb EF-G2 (Supplementary Figure S4). The G1 motif (Walker A motif) forms a P-loop that interacts with the α- and β-phosphates of GTP or GDP. The G3 motif (Walker B motif) forms the switch II region that interacts with the γ-phosphates of GTP and binds a water-bridged Mg^2+^ ion. The G4 motif mainly engages in interactions with the guanine (Seshadri et al., 2009). However, the G2 motif forming the switch I region and the G5 motif are not well conserved in Mtb EF-G2. The conserved RGITI motif is replaced by QQRSV in the G2 motif and the GSAF motif is replaced by VCSS in the G5 motif in Mtb EF-G2 (Supplementary Figure S4). The additional G′ subdomain is not very well conserved amongst various EF-Gs and EF-G2 in Mtb (Supplementary Figures S3, S4). However, some of the functionally important residues (E221, E225, and E228) have been shown to interact with the L7/L12 stalk of the 50S subunit in the G′ subdomain, and are mostly conserved between EF-G in various organisms and EF-G2 in Mtb (Supplementary Figures S3, S4).(Nechifor et al., 2007; Nechifor and Wilson, 2007; Seshadri et al., 2009). Domain II is a β sandwich domain built up of 11 β sheets (β13-β23).Two strands (β18-β19)extend from the barrel to the G domain, very close to the invisible effector region (Figure 1C; Supplementary Figure S5B, colored cyan). Interestingly, domain II of Mtb EF-G2 contains an insert of 17 residues not present in Mtb EF-G1. Residue R334 in the consensus motif of domain II is highly conserved among EF-G1 and EF-G2 in Mtb, and it forms salt bridge with D103 in the G-domain (Supplementary Figure S4).

Domain III comprises four antiparallel β-sheets (β24, β25, β26, β27) with two α helices on one side of the β-sheet (α9 and α10) and bridges between domains II and IV (Figure 1C; Supplementary Figure S5C, colored green). Domain III in different species is flexible, as is the case for the switch I and switch II regions, which indicates that domain III may be of functional importance. Domain IV is an elongated fold at the end of EF-G1. It contains two parallel β-sheets (β32, β33) connected by an α helix (α11) displaying an unusual left-handed structure (Figure 1C; Supplementary Figure S5D, colored yellow). Residue H580 of domain IV, which has been shown to play an important role in promoting tRNA movement, is conserved in Mtb EF-G1 and EF-G2 (Supplementary Figure S4) (Savelsbergh et al., 2000). The small β-sheets (β29, β34) connect domains III and V to domain IV, respectively. Finally, domain V has a double-split βαβ fold (Figure 1C; Supplementary Figure S5E, colored red).

Two interesting parts (switch I and switch II) of the Mtb EF-G1 G domain in molecule A are missing as a result of structural disorder. However, we could model switch II of Mtb EF-G1 in molecule B following refinement. This indicates that when GDP is bound, a large conformational change occurs in the two regions, or that these two regions are flexible and this may be important for EF-G function.

### The Active Site for GDP Binding

**FIGURE 2:**
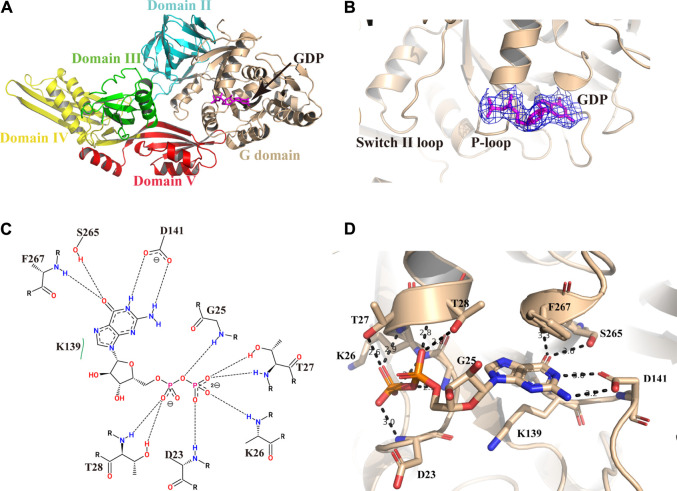
Structure of Mtb EF-G1 in complex with GDP. **(A)** Cartoon representation of Mtb EF-G1 colored by different domains. The docking of GDP in the active site is shown as a stick model in magenta. **(B)** A magnified view of GDP in the active site of Mtb EF-G1, with the final electron density map (2Fo-Fc, contoured at 0.8 σ) superimposed. **(C)** Detailed two-dimensional diagram of the active site of Mtb EF-G1 occupied by GDP. Directed bonds between Mtb EF-G1 and GDP are drawn as dashed lines. Image generated by PoseView (https://www.zbh.uni-hamburg.de/en/forschung/amd/server/poseview.html). **(D)** Ribbon model of the GDP binding pocket containing GDP. Residues recognizing GDP are shown in stick model. Hydrogen bonds between Mtb EF-G1 and GDP are indicated by dashed lines.

We pre-incubated GDP and/or GTP with the Mtb EF-G1 protein in a molar ratio of 5:1 prior to crystallization trials. After extensive efforts, we obtained crystals of the EF-G1●GDP complex. However, we were unable to crystallize the EF-G1●GTP complex. As illustrated in Figure 2A, the GDP molecule is bound in the G domain. The electron density for the GDP is well defined in the final map (Figure 2B). The Walker A motif (^23^DAGKTT^28^) forms the A-loop (P-loop), which mainly binds the α- and β-phosphates through main chain atoms in the same way as it does in other EF-G proteins (Czworkowski et al., 1994; Al-Karadaghi et al., 1996) (Figures 2C,D). The O3B, O2B, and O1B atoms of the β-phosphate are specifically recognized by the backbone NH groups of T27, K26, and D23 through multiple hydrogen bonds. The O1A of the α-phosphate is specifically recognized by two hydrogen bonds donated by T28 (Figures 2C,D). The absence of the ε amino group of the K26-β phosphate in the GDP hydrogen bond but the presence of the ε amino group of the K26-hydroxyl group in the T85 hydrogen bond is also observed in Mtb EF-G1. Therefore, EF-G has a low affinity for GDP. Additionally, we did not observe the presence of Mg^2+^, even when MgCl_2_ was added during crystallization.

The ribose part of GDP is only recognized by K139 through hydrophobic contacts. The guanosine base is surrounded by several hydrogen bonds donated by S265, F267, and D141. The HN1 and HN2 of the guanosine base accept two hydrogen bonds from the side chain of D141. The O6 atom of the guanosine base accepts two hydrogen bonds from the S265 side chain and the backbone NH group of F267 (Figures 2C,D).

The switch I region of Mtb EF-G1, essential for coupling GTP hydrolysis, was found to be disordered, which is consistent with previous study indicating that the switch I region of EF-G1 has not been modeled (Czworkowski et al., 1994; Hansson et al., 2005; Gao et al., 2009), except those in the EF-G in GTP form bound to a ratcheted ribosome and in the *S.aureus* EF-G structure (Chen Y. et al., 2010; Chen et al., 2013). Therefore, Mtb EF-G1 is inactive, and upon ribosome and GTP binding, switch I of Mtb EF-G1 may be stabilized as the structure of a pretranslocational ribosome bound to EF-G trapped with a GTP analog (Chen et al., 2013). The switch II region of EF-G, which is important in coupling transition from the GDP-bound to the GTP-bound conformation, is ordered in the Mtb EF-G1 structure. It was previously reported that the largest conformational changes between GDP-bound and GDP-unbound EF-G1 occur in the switch II region (Al-Karadaghi et al., 1996). By superimposing the two EF-G without GDP fragment crystal structures (PDB id:5TY0 and 5VH6) with Mtb EF-G1●GDP, we could see that the conformations of the backbones and side chains between G domain and domain II are very similar (Supplementary Figure S6A). In addition, the side chains of key residues constituting the GDP binding site have the same orientations as those in Mtb EF-G1●GDP (Supplementary Figure S6B). The differences between these three EF-G structures are conformational changes of the switch II and P-loop regions (Supplementary Figure S6C). These conformational changes probably result from GDP binding.

### Comparison of Mtb EF-G1 Structure With Previous EF-G Structures

**FIGURE 3:**
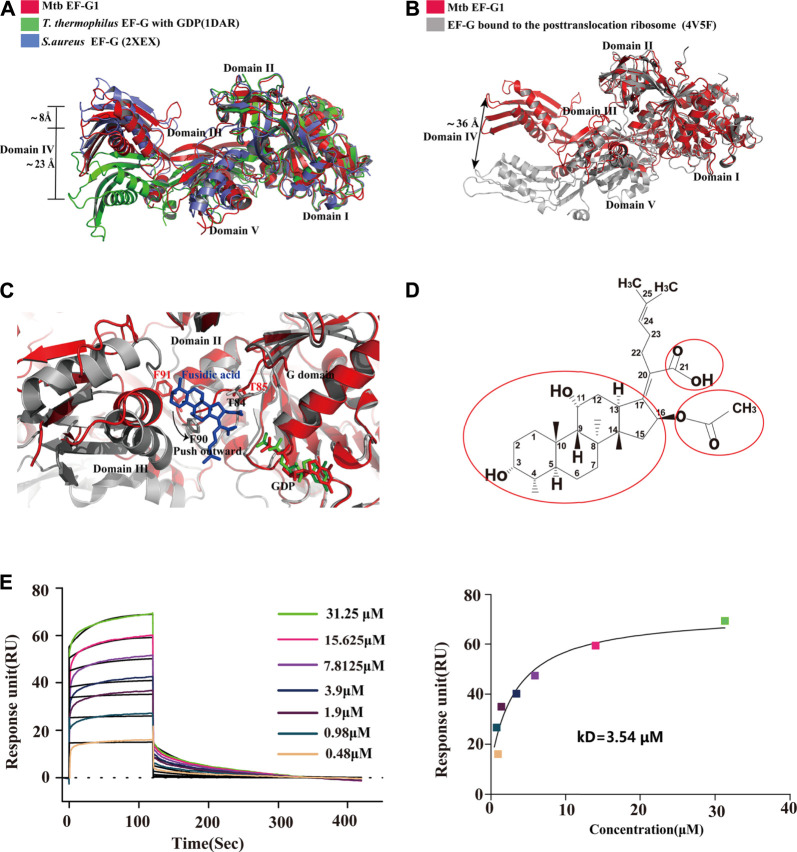
Comparison of the structures of Mtb EF-G1 with EF-G from other species and EF-G trapped in the post-translocational ribosome. **(A)** Comparison of Mtb EF-G1 with *T.thermophiles* EF-G (PDB id:1DAR) and *S.aureus* EF-G (PDB id:2XEX) revealing movement of the tip of domain IV between Mtb EF-G1 and *T.thermophiles* EF-G, and between Mtb EF-G1 and *S.aureus* EF-G. Superposition was achieved by aligning domains I and II of the two EF-G structures. Domains of EF-G are labeled with latin numbers. **(B)** Mtb EF-G1 (red)and EF-G bound to the post-translocational ribosome (gray, PDB id:4V5F) superposed by structural alignment of domains I and II. The arrow indicates the shift of the tip of domain IV between the two structures. **(C)** Superposition of domains I and II between the fusidic acid-bound structure (gray,PDB id:4V5F) with Mtb EF-G1 indicates movement outward or downward of the switch II region between the two structures. **(D)** Chemical structure of FA. The essential groups for activity are marked with red circles. **(E)** SPR analysis of FA binding to Mtb EF-G1(left)FA at various concentrations were injected over immobilized Mtb EF-G1. (right) Fitting curve for equilibrium binding that produced a kD of 3.54 μM.

To date, available data indicate that EF-G assumes different conformations that are mainly characterized by the movement of domains III, IV and V relative to domains I and II and no alternations in domains I and II. To gain insight into the structural differences between single domains of EF-G from different sources, we first compared Mtb EF-G1 with the previously isolated *T. thermophilus* EF-G bound to GDP, and the apo structure of *S.aureus* EF-G. The results showed that the individual domains (I-V) are highly similar. However, domains III, IV, and V display high variability relative to domains I and II (Figure 3A), and domains III-V are rotated relative to domains I-II, resulting in a movement of the tip of domain IV differing by 8 Å between our structure and apo *S.aureus* EF-G (Figure 3A), we examined the equivalent position displaying a movement by ∼23 Å between our structure and that of *T.thermophilus* EF-G bound to GDP (Figure 3A). Furthermore, comparison with the crystal structure of the ribosome with EF-G trapped in the post-translocational state reveals a significant structural rearrangement of domains III, IV, and V, resulting in a shift of the tip domain IV by up to ∼36 Å (Figure 3B). This distance is larger than that previously reported for *T.thermophilus* EF-G as shown by comparisons between apo EF-G and ribosome-bound EF-G (Chen Y. et al., 2010), these movements are consistent with previous results that EF-G undergoes interdomain rearrangement during the translocation cycle (Gao et al., 2009; Chen Y. et al., 2010; Brilot et al., 2013; Salsi et al., 2015; Ling and Ermolenko, 2016).

### Proposed FA Binding Site of Mtb EF-G1 and Future Chemical Modification of FA

FA is a natural steroid antibiotic that has been used clinically for the treatment of Gram-positive infections and methicillin-resistant *S.aureus* (Whitby, 1999; Sahm et al., 2013) . FA has also been shown to exhibit activity *in vitro* against Mtb (Cicek-Saydam et al., 2001). FA specially inhibits EF-G and locks it onto the ribosome after GTP hydrolysis and translocation. Because the EF-G-FA structure remains unavailable, we modeled binding of FA to Mtb EF-G1 by superimposing our structure onto the structure of EF-G bound to the *T. thermophilus* 70S ribosome with GDP and FA (PDB id: 4V5F) based on domains I and II. FA binds to a pocket surrounded by domains I, II, and III of Mtb EF-G1, similar to the *T. thermophilus* 70 S ribosome with GDP and FA (Figure 3C). Further comparison of the structure of Mtb EF-G1 and the EF-G–70S complex with GDP and FA indicates that binding of FA induces evident conformational changes in switch II, which includes movement of switch II outward or downward, relative to the corresponding switch II Mtb EF-G1 structure (Figure 3C). Two EF-G mutations at T85 and F91 in switch II caused a shift of 3–5 Å, resulting in a direct interaction with FA. In other words, FA binding prevents the switch II region from adopting its GDP conformation, and locks it in a conformation similar to that of the GTP form (Gao et al., 2009). Modeling of FA on Mtb EF-G1 indicated that the *trans-syn*-*trans* conformation of the tetracylic triterpene backbone, the carboxylic acid (C-21), and acetoxy (C-16) groups of FA occupy a hydrophobic cavity on EF-G, and all are indispensable for inhibitor design, consistent with the structure-activity relationship (SAR) (Figures 3C,D). In addition, the orientation of the lipophilic side chain and the carboxyl group around the Δ17,20 bond, rather than the double bond, are essential for anti-mycobacterial activity, and should be considered in future inhibitor design (von Daehne et al., 1979; Duvold et al., 2001). To validate our model, we employed SPR to investigate the binding affinity of FA to Mtb EF-G1. The binding affinity of Mtb EF-G1 with FA determined by our SPR experiments exhibited a kD of 3.54 μM (Figure 3E), which falls in with the range of kD values of 4.34–8.33 μM reported previously for *E.coli* EF-G using equilibrium dialysis (Okura et al., 1970; 1971) . This result indicates that FA can indeed bind to Mtb EF-G1, explaining why FA is effective against Mtb *in vitro*. However, it is still disputed whether FA can directly bind to EF-G in the absence of ribosomes (Willie et al., 1975). Further verification of FA binding to EF-G from different organisms using various biophysical techniques and structural studies should finally resolve this dispute. To prevent FA from being rapidly metabolized *in vivo*, one group repurposed FA via chemical modification and performed biological characterization, and found that the antimycobacterial activity of C-3 silicate esters was comparable to that of FA, and these compounds were stable in microsomes and plasma, revealing them to be attractive compounds for *in vivo* antimycobacterial activity evaluation (Njoroge et al., 2019). Finally, The structure of the Mtb ribosome in complex with EF-G and FA derivatives (C-3 silicates) will offer valuable insight into the design of FA analog inhibitors targeting EF-G.

## Conclusion

In summary, we purified recombinantly expressed Mtb EF-G1 and characterized its GDP binding activity, and then provide the first crystal structure of Mtb EF-G1 in complex with GDP. Our unique crystal form provide an excellent platform for the fragment-based screening using crystallographic approach and will play an important role in design and development of enzyme inhibitors of potential interest for future studies in the context of TB drug discovery.

## Data Availability

The atomic coordinates and structure factors have been deposited in the Protein Data Bank (http://www.wwpdb.org) under the accession codes: 7CDW.
