# Supplementary material for: Crystal Structure of Mycobacterium tuberculosis Elongation Factor G1
Source: Front Mol Biosci. 2021 Sep 3;8:667638. doi: 10.3389/fmolb.2021.667638 (PMC8446442; doi:10.3389/fmolb.2021.667638)
Supplement: Supplementary file 1 [file DataSheet1.docx]

**Supplemental materials for**

**Crystal Structure of** ***Mycobacterium Tuberculosis* Elongation Factor G1**

**Supplemental figures**


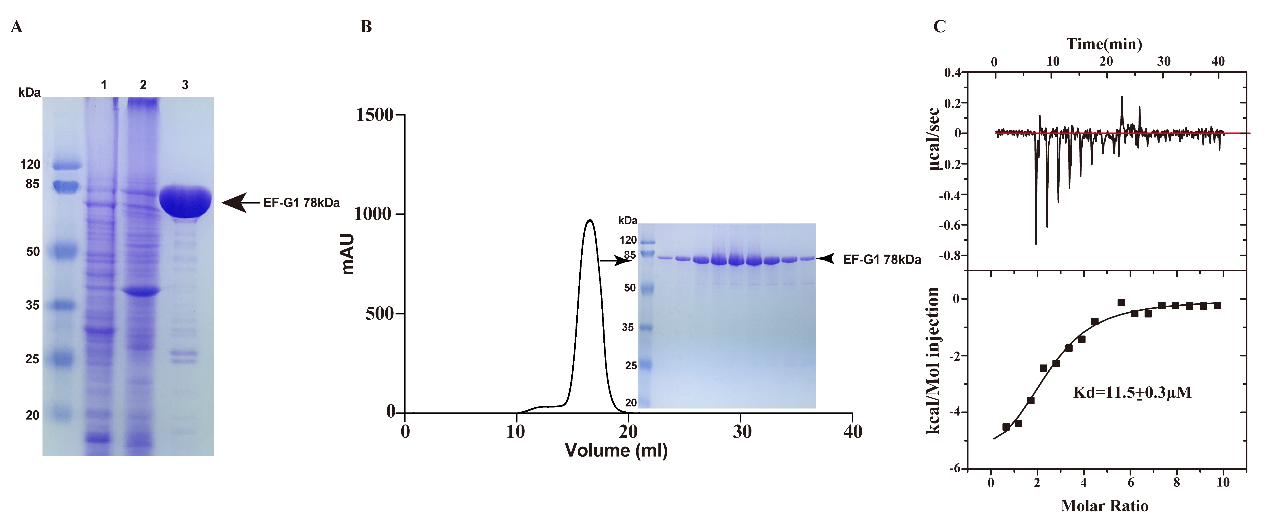


**Figure S1 Purification and biochemical characterizations of recombinant Mtb EF-G1.**

1. Preliminary purification of Mtb EF-G1. Lane1, supernatant of the bacteria lysis; lane 2, inclusion bodies from total lysate after centrifugation; lane 3, purified Mtb EF-G1 elution
2. Final purification of Mtb EF-G1 using gel-filtration chromatography. Right insert SDS-PAGE (12%) analysis of the peak fractions from the Superdex 200 10/300GL column.
3. Isothermal titration calorimetry analysis of the Mtb EF-G1 and GDP interaction. The raw titration curves (upper panels) and integrated binding isotherms (lower panels) for Mtb EF-G1 binding to GDP. The calculated *Kd* values are presented in the lower panels. The *Kd* values were showed from single measurements, and errors were denoted by curve fitting.

**
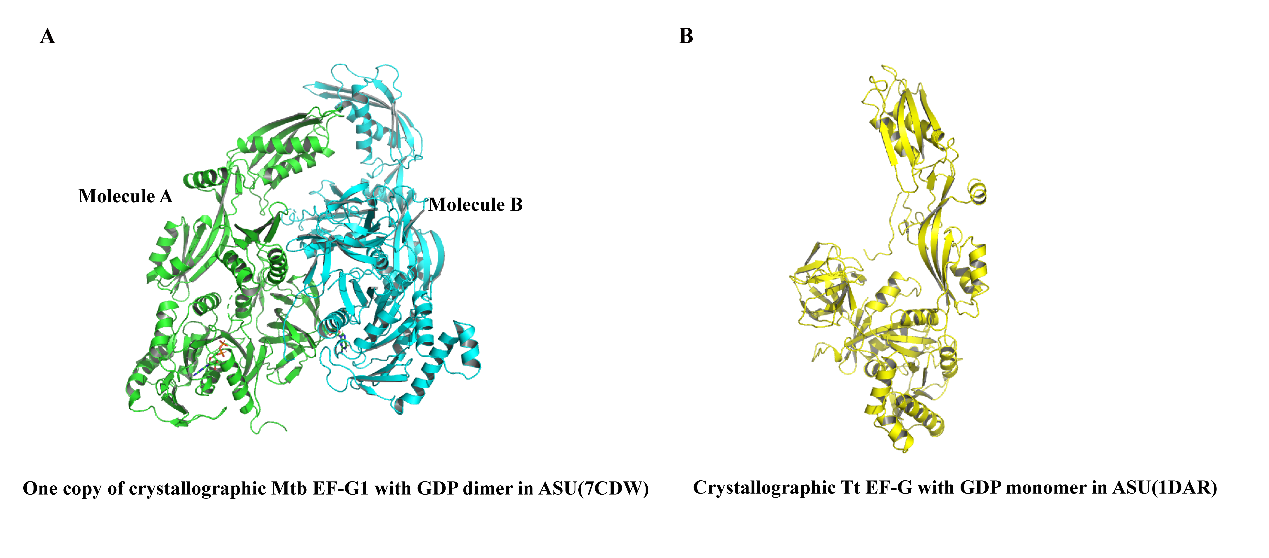
**

**Figure S2 Crystal packing analysis of the Mtb EF-G1 with GDP.**

1. Two molecules named A and B are present in the asymmetric unit of the crystal structure of Mtb EF-G1 with GDP
2. One crystallographic molecule (PDB id:1DAR) is present in the asymmetric unit of the crystal structure *Thermus thermophilus* (Tt)EF-G with GDP

**
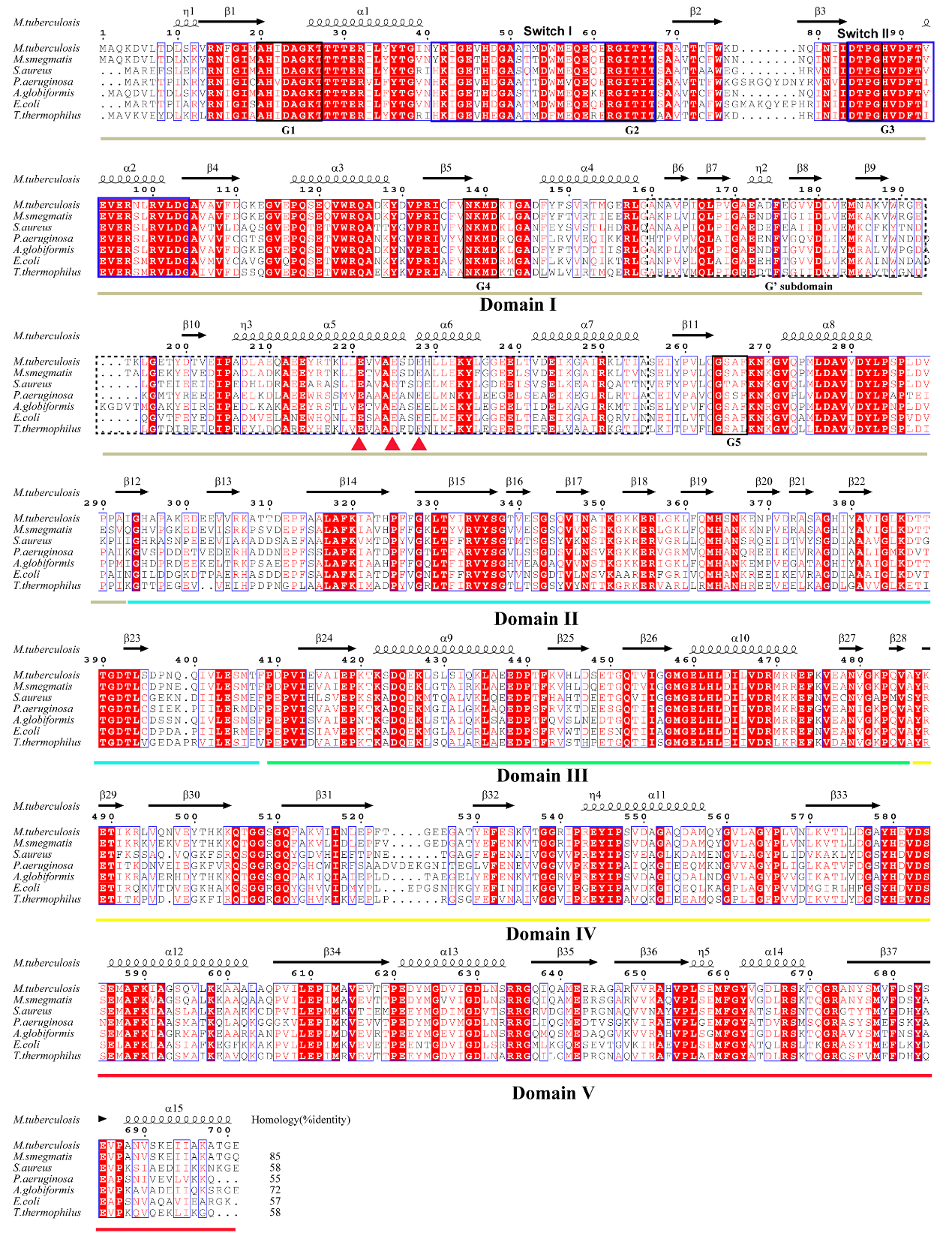
**

**Figure S3 Structure-based multiple sequence alignment of EF-G from different species.**

The multiple sequence alignment of EF-G from different species(*M.tuberculosis*,*M.smegmatis,S.aureus,P.aeruginosa,A.globiformis,E.coli*and *T.thermophilus*).Secondary structure elements of Mtb EF-G1 are represented on top of the sequences. Residues with the red background indicate invariant residues. The conserved GTPase motifs are boxed and numbered. The switch I and II region are marked with a blue box. The G’sub-domain is boxed with broken lines. The functionally important residues(E221, E225, and E228) in the G’ subdomain are indicated with a red-filled triangle. Conserved motifs (domain I-V)are indicated at the bottom of the sequences with different colors. The alignment was performed using the multiple sequence alignment program ClustalW and ESPript 3.0. The homology (%identity) of each protein is compared with Mtb EF-G1 by performing with DNAMAN software and shown at the end of each sequence.

*
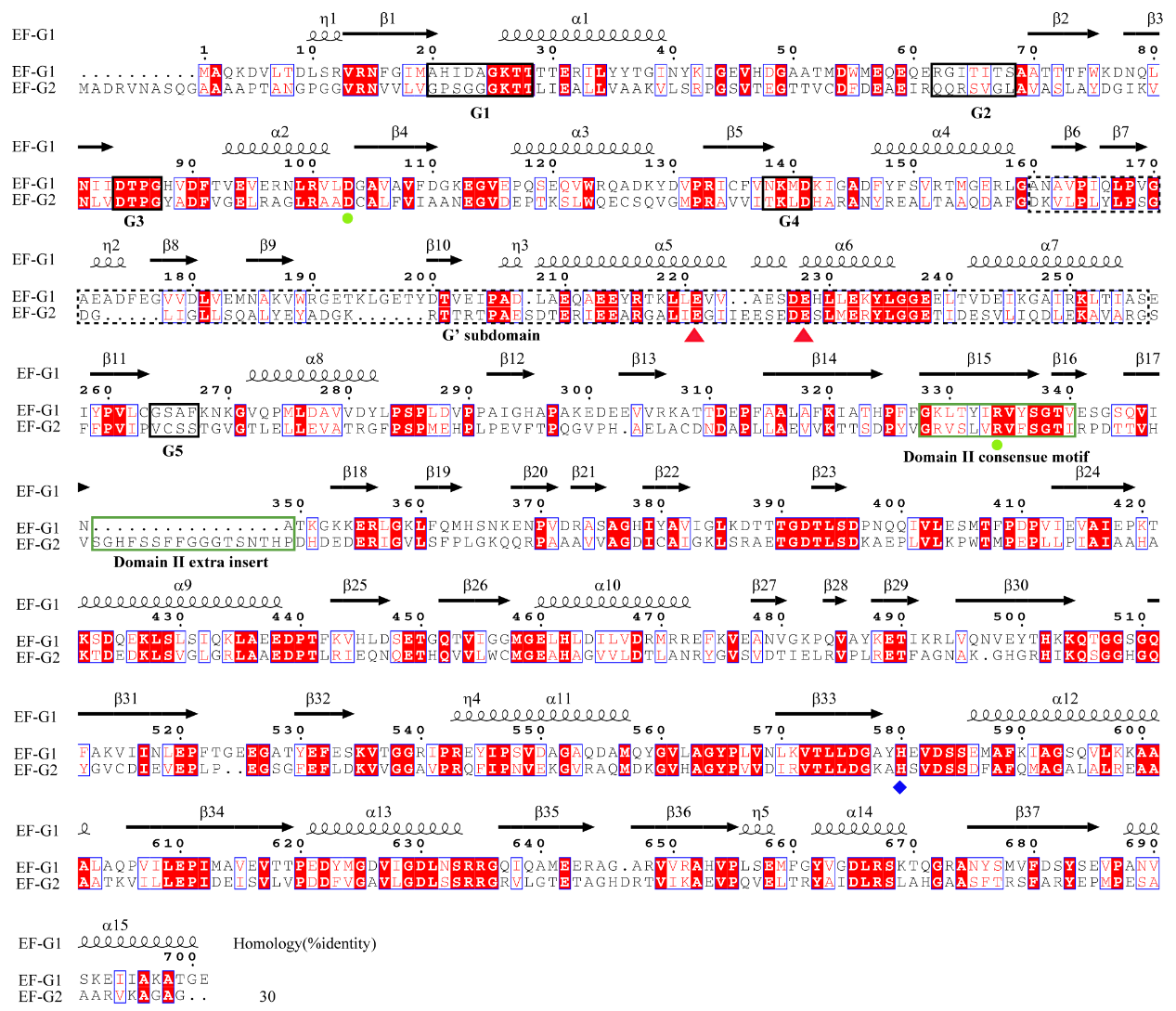
*

**Figure S4 Structure-based multiple sequence alignment of EF-G1 and EF-G2 from *M.tuberculosis.***

The sequence alignment of EF-G1 and EF-G2 from *M.tuberculosis*. Secondary structure elements of Mtb EF-G1 are shown at the top. Invariant residues are shown with red background and conserved residues are shown in red fonts. The G domain including G1-G5 is boxed and numbered. The G’sub-domain is also boxed with broken lines. The conserved domain II motif and domain II extra insert are boxed and indicated at the bottom of the sequences. Other conserved residues are indicated with filled circles and filled diamonds.The sequence alignment was calculated and rendered using the software Clustal Omega and ESPript 3.0. The homology (%identity) between EF-G1 and EF-G1 is compared by performing with DNAMAN software and shown at the end of each sequence.

**
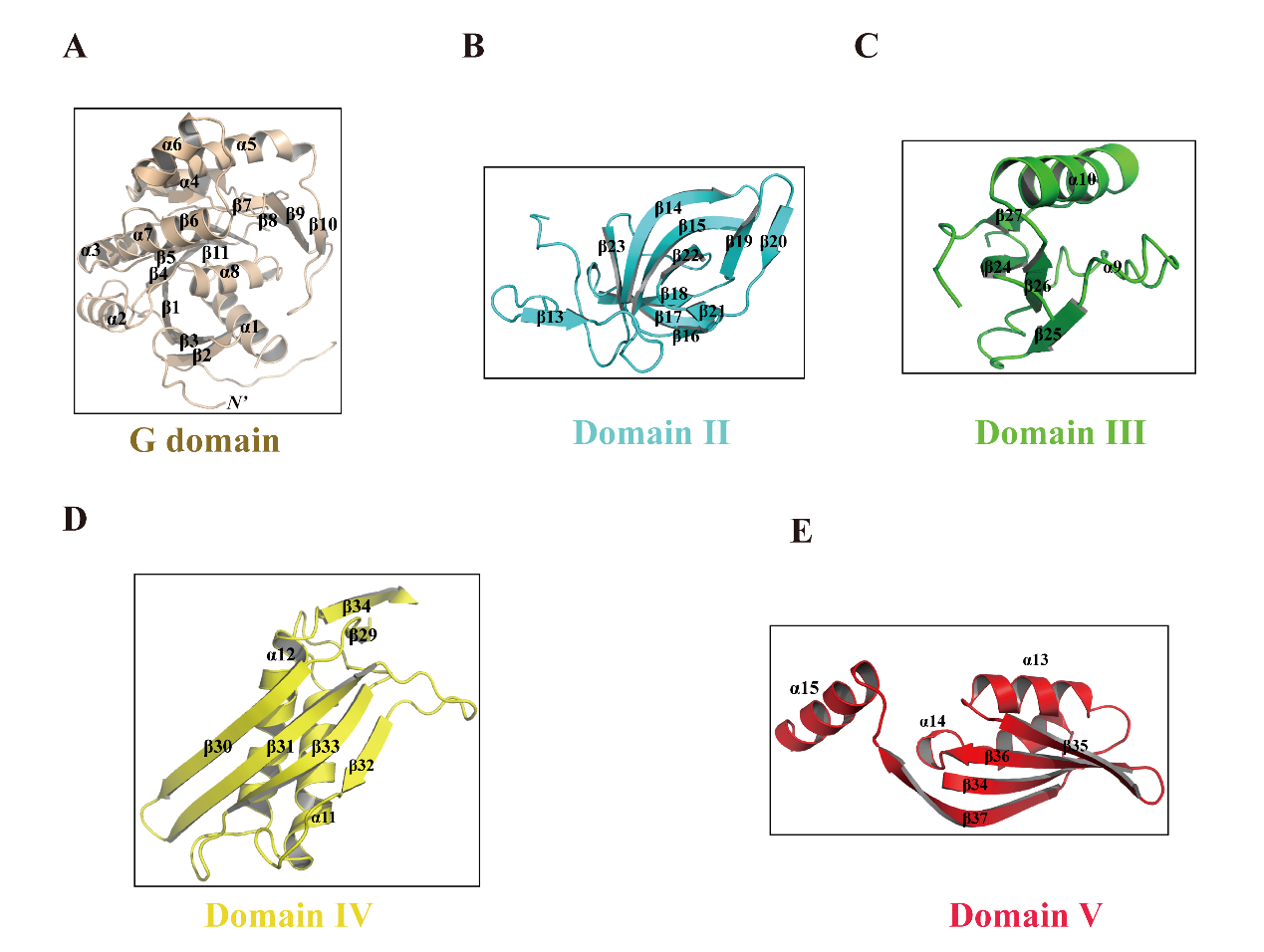
**

**Figure S5 The individual domain of Mtb EF-G1.**

The ribbon cartoon of the Mtb EF-G1 G domain(A), domain II(B), domain III(C), domain IV(D) and domain V(E).

**
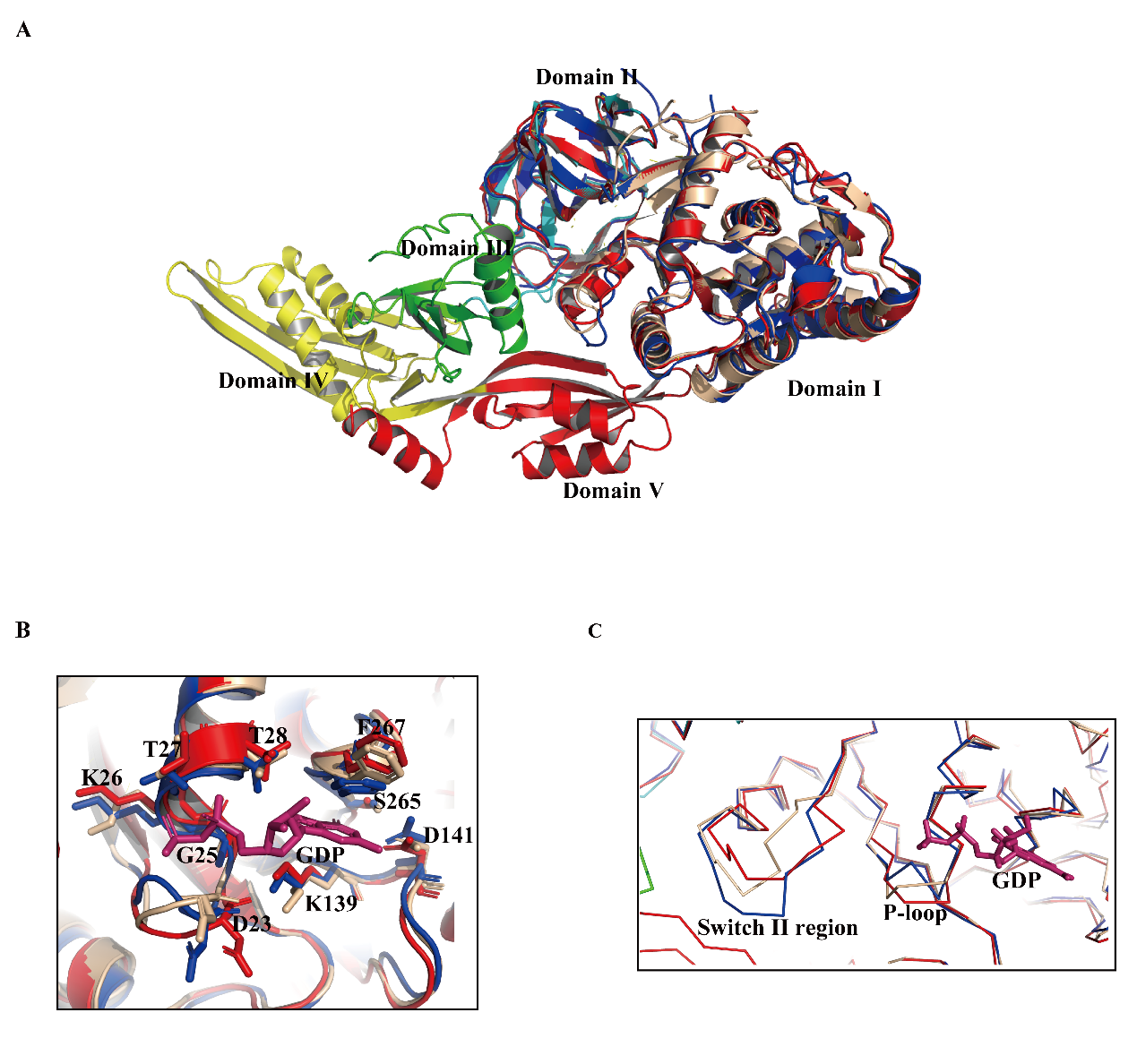
**

**Figure S6 Comparison of the structures of Mtb EF-G1-GDP with other species EF-G.**

1. Superimposition of Mtb EF-G1●GDP with *Legionella pneumophila* EF-G (red, PDB: 5TY0) and *Bacillus subtilis* EF-G (blue, PDB: 5VH6) . Mtb EF-G1 is colored by subdomains.
2. Superimposition of the Mtb EF-G1 bound GDP (warmpink) with that of *Legionella pneumophila* EF-G (red, PDB: 5TY0) and *Bacillus subtilis* EF-G (blue, PDB: 5VH6) active sites. The conserved residues involved in GDP binding are shown as stick models.
3. Superimposition of the Mtb EF-G1●GDP switch II region with those of *Legionella pneumophila* EF-G (red, PDB: 5TY0) and *Bacillus subtilis* EF-G (blue, PDB: 5VH6).

**Supplementary Tables**

**Tables S1** **Data collection and refinement statistics**

|  | **Mtb EF-G1**  **expressed**  **Se-Met crystal**  **(PDB ID:7CDW)** |
| --- | --- |
| **Data collection** |  |
| Space group | C222_1_ |
| Cell dimensions |  |
| a, b, c (Å) | 101.54 301.52 145.87 |
| α, β, γ (°) | 90.00, 90.00, 90.00 |
| Matthews coefficient (Å^3^/Da) | 3.58 |
| Solvent Content (%) | 65.6 |
| X ray source |  |
| Wavelength (Å) | 0.97931 |
| Data range (Å) | 48.86-3.00 |
| Reflections unique | 83429^a^ |
| *R*_sym_ ^b^ (last shell) | 0.092 (0.66) |
| *I* / σ*I* (last shell) | 11.73 (2.37) |
| CC_1/2_ (%) | 99.6(81.2) |
| Completeness (%) (last shell) | 96.7 (97.6) |
| Redundancy (last shell) | 2.92 (2.88) |
| SigAno ^c^ | 1.40 |
| **Refinement** |  |
| Resolution range (Å) | 47.95-3.00 |
| Reflections  cut-off, cross validation | 83392^a^  F>1.33, 3800 |
| *R*_work_ ^d^/ *R*_free_ ^e^ (last shell) | 0.2062/0.2414 (0.3275/0.3352) |
| **Atoms** |  |
| Non-hydrogen protein atoms | 10537 |
| Protein | 10537 |
| Solvent | 0 |
| *B*-factors average (Å^2^) | 68.92 |
| Protein (Å^2^) | 69.01 |
| Ligands (Å^2^) | 51.50 |
| Solvent (Å^2^) | 0 |
| **r.m.s.d** |  |
| Bond lengths (Å) | 0.004 |
| Bond angles (°) | 0.841 |
| % residues in favored regions, allowed regions, outliers in Ramachandran plot | 97.63,2.37,0 |

^a^ Friedel pairs are treated as different reflections

^b^ *R*_sym_ = ∑_hkl_∑_j_ |I_hkl,j_ - I_hkl_|/∑_hkl_∑_j_I_hkl,j_, where I_hkl_ is the average of symmetry-related observations of a unique reflection.

^c^ SigAno(overall) : mean anomalous difference in units of its estimated standard deviation (|F(+)- F(-)|/Sigma). F (+), F (-) are structure factor estimates obtained from the merged intensity observations in each parity class.

^d^ *R*_work_ = ∑_hkl_ ||*F*_obs_(hkl)|-|*F*_calc_(hkl)||/∑_hkl_|*F*_obs_(hkl)|.

^e^ *R*_free_= the cross-validation *R* factor for 5% of reflections against which the model was not refined.
